# Dengue Burden and Circulation of Dengue-2 Serotype Among Children Along With Clinical Profiling in Uttarakhand, India: A Cross-Sectional Study From 2018 to 2020

**DOI:** 10.7759/cureus.33913

**Published:** 2023-01-18

**Authors:** Gaurav Badoni, Pratima Gupta, Manju O Pai, Neelam Kaistha, Radhakanta Ratho, Nusayha Sokeechand

**Affiliations:** 1 Microbiology, All India Institute of Medical Sciences, Rishikesh, Rishikesh, IND; 2 Microbiology, All India Institute of Medical Sciences, Deoghar, Deoghar, IND; 3 Molecular Biology, Proteomics and Metabolics, All India Institute of Medical Sciences, Rishikesh, Rishikesh, IND; 4 Virology, Postgraduate Institute of Medical Education and Research, Chandigarh, Chandigarh, IND; 5 Biostatistics, Centre International de Développement Pharmaceutique, Phoenix, MUS

**Keywords:** reverse transcriptase polymerase chain reaction (rt-pcr), denv-2, child and adolescent, dengue virus serotype, vector-borne disease

## Abstract

Background: The multiple serotypes of the dengue virus (DENV) are always a major public concern for dengue haemorrhagic fever (DHF) and dengue shock syndrome (DSS).

Objective: This study aimed to analyse the demographic characteristics and circulating serotypes of dengue among the paediatric age group.

Methodology: One hundred forty-one clinically suspected children were enrolled in the study from 2018 to 2020 in a tertiary care hospital in Uttarakhand, India. Central tendency, frequency, and One-Way ANOVA were measured for continuous and categorical data. The Shapiro-Wilks test was used to calculate the normality assumption. Dengue NS1 Ag, IgM, and IgG antibodies ELISA were performed, and NS1-positive samples were further tested for molecular studies.

Result: From one hundred forty-one suspected cases, 100 (70.92%) came positive for dengue NS1 antigen, 18 (12.76%), and three (2.12%) came positive for IgM and IgG antibodies respectively. Rest 20 (14.18%) samples came negative for dengue. Fever with chills (97.5%), headache (89%), and arthralgia (82%) were the most common clinical features. Molecular studies showed the dengue serotype-2 (DEN-2) was found in most cases, followed by the dengue-3 serotype (DEN-3).

Conclusion: This is the preliminary study as the authors' best knowledge which demonstrate the burden of dengue in children with prevalent serotypes for consecutive three years in Uttarakhand. This study identifies that the serotype-2 (DEN-2) of the dengue virus was the primary cause of infection in children at the tertiary care hospital in northern India. These results will help further to understand the nature of the disease so that improved patient care management will imply. Further molecular studies on large sample sizes during the endemic would be helpful to gain knowledge of the actual load of the disease and the genetic characteristics of the virus.

## Introduction

Dengue is a major public health disease globally with approximately 5.2 million cases in 2019 [1]. The total number of cases and reported deaths appear to have decreased during the year 2020. However, the data are insufficient, and the COVID-19 pandemic may have hampered case reporting in several countries [2]. In the Region of Americas, a total of 2,459,455 cases of arboviral infection were reported in 2020, out of these cases, 2,333,508 (94.9%) were dengue [3]. Dengue virus (DENV) belongs to the Flaviviridae family and is transmitted by mosquitoes, especially from Aedes aegypti which is a domestic daytime feeder mosquito [4]. Dengue virus has four serotypes i.e., DEN-1 to 4 and the infection can be caused by any of these four serotypes. An individual develops lifelong immunity to one serotype after infection but the following infection with another serotype can improve the risk of severe dengue due to antibody-dependent enhancement of infection [5]. Dengue serotype 2 has been the most common serotype over the last 50 years, but serotypes 3 and 4 have also been seen in some epidemics [6]. For the Indian scenario, approximately 0.8 million dengue cases have been recorded from 2017 to 2022 followed by 1,000 deaths [7]. Maximum cases of dengue have been recorded in 2021 followed by 2019.

Uttarakhand is the 27th state of India, situated in the foothills of the Himalayas and surrounded by the international border of China and Nepal. In Uttarakhand, more than 16,000 dengue cases have been reported from 2017 to 2022 [7]. As the Uttarakhand state is well known for pilgrims and tourists not only from India but from foreign also, it becomes necessary to identify the serotypes of dengue virus in various locations of this state for early calculation of the disease and establish the required patient care management accordingly.

To the lack of information about dengue in children from the region, we attempt to estimate the burden of the dengue virus among children, as well as clinical manifestations, and try to identify any changes in the circulating dengue serotypes during three consecutive years.

## Materials and methods

Study design, site, and period

This cross-sectional exploratory study was conducted in a tertiary care hospital in Uttarakhand, India, from 2018 to 2020 which is situated in the foothills of the Himalayas at 30.0668° N, and 79.0193° E.

Patient recruitment and data collection

The data on circulating serotypes of the dengue virus among children in the Uttarakhand state was unknown. Hence, a cross-sectional exploratory study was conducted from 2018 to 2020 at AIIMS Rishikesh, India. Inclusion: i) Children (≤17 years); ii) showing acute febrile illness defined as fever >38°C from one to 14 days, falling in a suspected case of dengue with or without warning signs as defined by WHO/IDSP; iii) participants or their parents/guardian provide written consent. Exclusion: i) Participants more than 17 years of age; ii) showing signs of upper respiratory tract infection; iii) incomplete data; iv) refusing to give written informed consent. The research was carried out under the World Medical Association's Helsinki Declaration and was approved by Institutional Ethics Committee, AIIMS Rishikesh (IEC: 46/IEC/PhD/2018).

A sample size of 136 children was calculated by the prevalence of NS1-positive children from the previous year which was 9.8% and 5% margin of error at a 95% confidence interval using R software. We chose to take all 141 (11%) children for this study from a total of 1,295 dengue-suspected participants in AIIMS Rishikesh from 2018 to 2020 to increase the power of the analysis. Because dengue fever is a seasonal disease, the samples were collected each consecutive year during the monsoon and post-monsoon seasons. Approximately 3 ml blood samples were collected only once at the time of enrolment. Samples labelled with test id, were centrifuged, and serum was stored at -80˚C for further investigation. Detailed clinical history including demographical information with informed consent was taken before collecting the samples.

All samples were tested for the dengue virus NS1 ELISA kit (EUROIMMUN, USA), and anti-dengue virus ELISA (IgM and IgG) kit (EUROIMMUN, USA) as per the kits’ manual. The observance was assessed at 450nm, and analysis was done as described by the manufacturer. NS1-positive samples will be considered DENV-positive in the study and further analysed for molecular studies.

RNA isolation and synthesis of cDNA

150 µL NS1 positive serum samples were performed for RNA isolation by using Macherey-Nagel™ NucleoSpin™ RNA, Mini Kit (Germany) according to the kits’ manual. Obtained RNA was kept at -80˚C for further analysis. cDNA synthesis was carried out from stored extracted RNA. 20 µL cDNA synthesis from 10 µL of RNA was done from a High-capacity cDNA Reverse Transcription kit (Applied Biosystems).

Detection of dengue virus by PCR

PCR amplifies the CprM junction by the method of Lanciotti et al. [8] with some modifications. cDNA was first amplified with dengue virus upstream consensus primer (D1: 5’-TCAATATGCTGAAACGCGCGAGAAACCG-3’) and downstream consensus primer (D2: 5’- TTGCACCAACAGTCAATGTCTTCAGGTTC-3’) homologous to the genomic RNA of the four serotypes dengue virus with 10x PCR buffer, 25mM MgCl2, 20mM dNTPs, Taq polymerase in thermocycler of 25 µL final volume for initial denaturation of cDNA at 95˚C for 2 minutes followed by 40 cycles at 94˚C for 30 s, 58˚C for 1 min, 72˚C for 2 min. The final extension was conducted at 72˚C for 10 min. The product was observed on 1.5% agarose gel electrophoresis.

Identification of dengue virus serotypes:

Amplified product from D1 and D2 consensus primer was further purified and analysed for dengue virus serotypes based on Lanciotti et al. primers [8]. Semi-nested PCR was used to identify DENV serotypes. Types specific primers [8] with upstream consensus primer (D1) were added in amplified product with 10x PCR buffer, 25mM MgCl2, 20mM dNTPs, and Taq Polymerase. The total mixture makes up to 25 µL by adding nuclease-free water in a single tube. Initial denaturation was performed at 95˚C for 2 min followed by 40 cycles at 94˚C for 30 s, 55˚C for 1 min, and 72˚C for 2 min. The final extension was performed at 72˚C for 10 min. The amplified product from the above RT-PCR was electrophoresed on a 1.5% agarose gel. 

Statistical analysis

Measure of central tendency, frequency, and One-Way ANOVA were used for continuous and categorical data. Shapiro-Wilks test was used to calculate the normality assumption. R version 4.1.2 (R Foundation for Statistical Computing, Vienna, Austria) and SPSS software (IBM Corp. Released 2020. IBM SPSS Statistics for Windows, Version 27.0. Armonk, NY: IBM Corp was used for statistical tests and graph plotting.

## Results

Demographic properties

**Figure 1:**
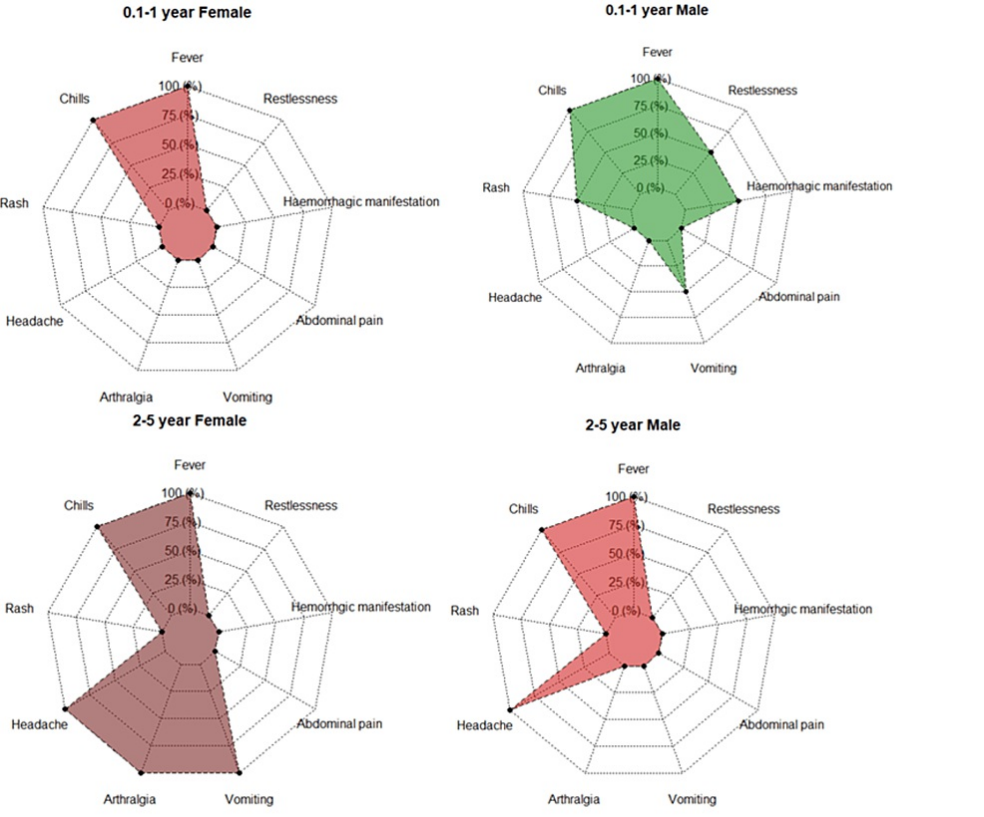
Clinical presentation of different age groups (0.1-5 years) of dengue-positive children.

**Figure 2:**
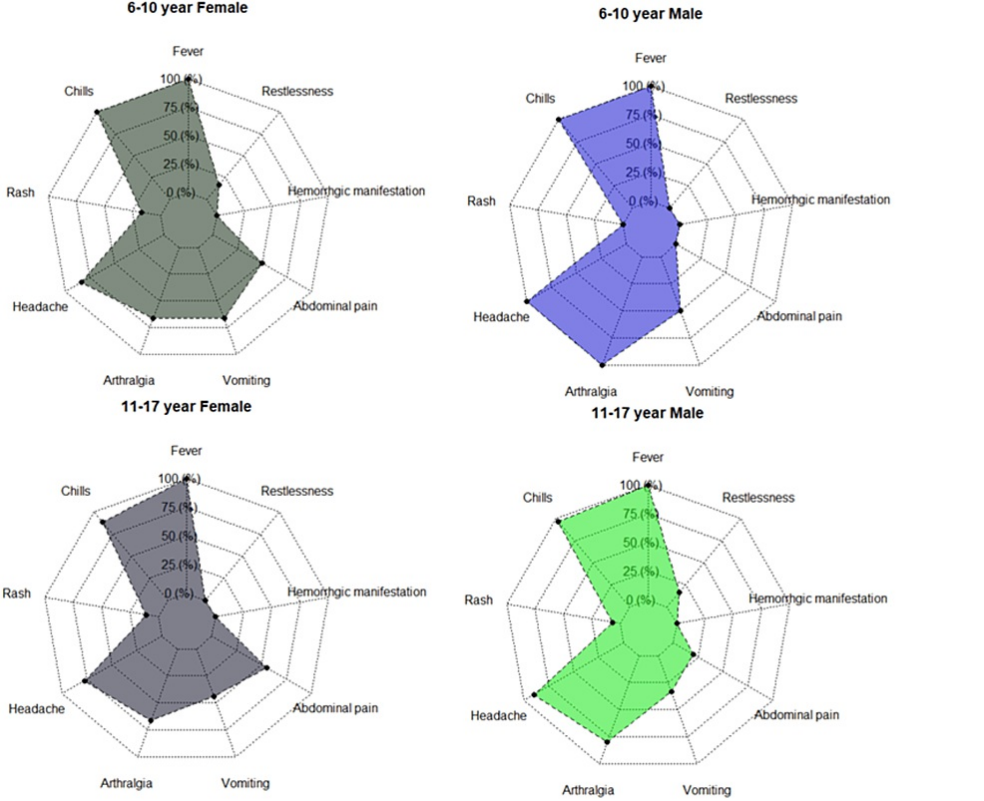
Clinical presentation of different age groups (6-17 years) of dengue-positive children.

**Figure 3:**
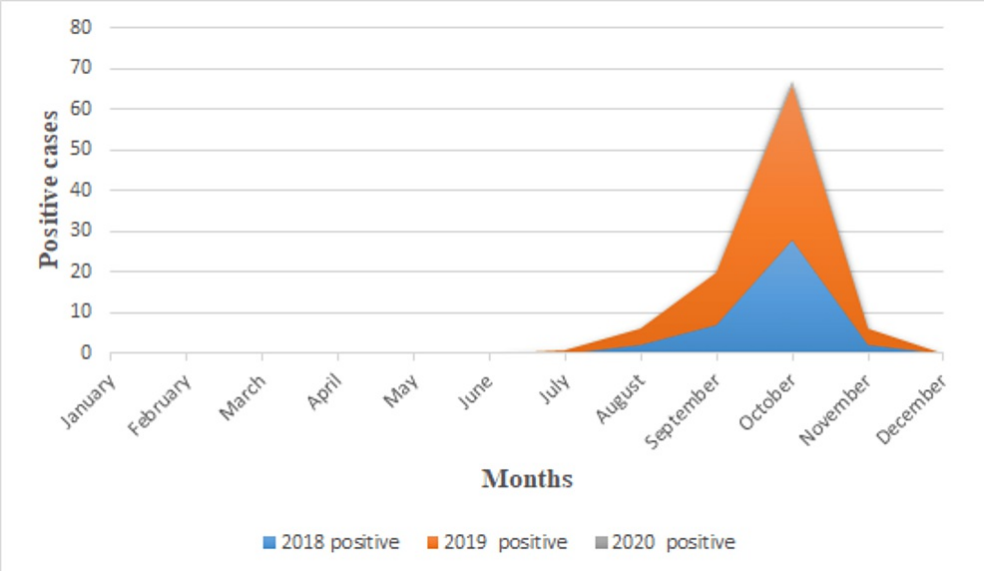
Month-wise distribution of dengue-positive cases from January 2018 to December 2020.

**Figure 4:**
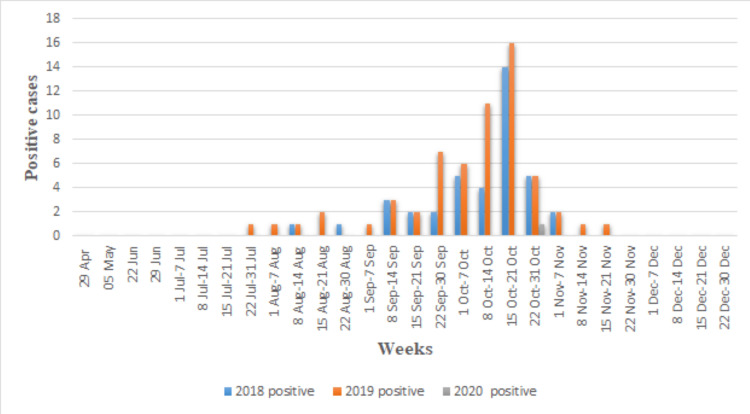
Week-wise distribution of dengue-positive cases from January 2018 to December 2020.

One hundred forty-one suspected clinical samples of children were screened for dengue infection from January 2018 to December 2020. Out of that, 100 (70.92%) came positive for dengue NS1 antigen, 18 (12.76%) for IgM and three (2.12%) for IgG. Rest 20 (14.18%) samples came negative for dengue. There were 68 (68%) males and 32 (32%) females among the 100 NS1 positives with M:F; 2.13:1. The difference between male and female preponderance among all positive cases was statistically significant (<0.0001) in 2018-19. In 2020, the number of patients in flaw was less due to the pandemic. Only 230 suspected cases, followed by 10 dengue-positive cases, were recorded in 2020. The different age and gender data showed that males are more outnumbered by females. The age group of 11-17 years was severely affected by dengue infection in 2018-2019 (Table 1). Most patients had fever with chills as common symptoms, followed by headache, vomiting, and arthralgia (Table 2, Figures 1, 2). Only five (5%) children had suffered from haemorrhagic manifestation, in which one (20%) of patients complained about epistaxis, three (60%) complained about haematemesis, and one (20%) had haematochezia. The highest positive rates (67%) and percentage of acute dengue cases in children (44.8%) were recorded in the second week of October from 2018 to 2020 (Figures 3, 4).

**Table 1:** Demographic properties of dengue-positive patients. *^a^*p-value corresponding to the one-sample chi-square test. The null hypothesis is that the proportion is equal across categories.

		2018 n= 39	2019 n= 60	2020 n=1
	Mean ± SD	11.2± 4.6	11.8± 4.2	1
Age Category	0.1-1	3	2	0
	2-5	1	3	0
	6-10	10	15	0
	11-17	25	40	1
	p-value^a^	<0.05 (S)	<0.05 (S)	NS
Gender	F	11	21	0
	M	28	39	1
	p-value^a^	<0.05 (S)	<0.05(S)	NS
	Age Category	2018 n= 39	2019 n= 60	2020 n=1
Female	0.1-1	0	0	0
	2-5	0	1	0
	6-10	7	8	0
	11-17	4	12	0
Male	0.1-1	3	2	0
	2-5	1	2	0
	6-10	3	7	0
	11-17	21	28	1

**Table 2:** Clinical symptoms of DENV-positive patients. u/k= symptoms are not known.

Clinical signs & symptoms	Age group			
	0.1-1 n=3, %	2-5 n=4, %	6-10 n=23, %	11-17 n=70, %
Fever	3, 100	4, 100	23, 100	69, 98.57
Chills	3, 100	4, 100	23, 100	66, 94.28
Rash	1, 33.33	0	1, 4.34	33, 47.14
Headache	u/k	3, 75	22, 95.65	64, 91.42
Arthralgia	u/k	1, 25	21, 91.30	60, 85.71
Vomiting	0	1, 25	18, 78.26	30, 42.85
Abdominal pain	u/k	0	16, 69.56	15, 21.42
Haemorrhagic manifestation	1, 33.33	0	0	4, 5.71
Restlessness	1, 33.33	0	1, 4.34	35, 50

Serotyping and gene sequencing analysis

51 samples were processed for serotyping identification. Out of that, 12 (23.52%) were positive for PCR, among that 11 (91.66%) were positive for DENV-2, and one (8.33%) for DENV-3. DENV-1 and 4 were absent from the tested samples (Table 3). Rest had insufficient viral load leading to negative PCR results.

**Table 3:** Analysis of dengue cases.

Sample	2018	2019	2020
Total sample	54	56	31
NS1 positive	39	60	1
Sample processed for PCR	20	30	1
PCR Positive	4 (20%)	7 (23.33%)	1 (100%)
DEN1	0	0	0
DEN2	3 (75%)	7 (85.71%)	1 (100%)
DEN3	1 (25%)	0 (14.28%)	0
DEN4	0	0	0

## Discussion

Dengue fever is a major public health concern in all tropical and subtropical regions around the world. The distribution of DENV serotypes and the increased likelihood of co-infection make the disease hyperendemic, with severe outbreaks occurring at regular intervals. In India, from 2017 to 2022, approximately 0.8 million dengue cases were reported including fourteen thousand cases from Uttarakhand [7]. Some studies from the past like the 2007 Haldwani city outbreak, 2009 outbreak, and 2012-2013 data from Dehradun city give us some information about the dengue situation of Uttarakhand but failed to provide serotypes of dengue in children [9-12].

During this study, we observed that the majority of dengue-positive children (85%) were coming from 11 to 17-year males. Post-monsoon season, i.e., September-October has the highest positive cases compared to the rest of the months. A study of 2013 by Singh et al. said that September month of 2013 documented the highest number of dengue cases [12]. In the present study, October has the highest (67%) positivity, and the second week of October recorded the maximum (44.8%) acute cases of dengue in children from 2018 to 2020. It is because of stagnation water in waste tyres, tin boxes, and empty pots after the monsoon. Some studies support this theory that poor rainwater management leads to accumulating water before and after the monsoon and gives mosquitoes favourable conditions to bread [10,11,13].

The clinical profile of dengue in children was similar to the previously reported studies. The maximum number of patients complained of fever with chills followed by headache and arthralgia. In a study by Ravikumar et al., fever (98%) was the most common clinical feature followed by vomiting (78%), hepatomegaly (68%), abdominal pain (68%), and edema (32%) the common clinical symptoms in children [14]. Bandyopadhyay et al. also state that 11-20-year males were most affected age during the 2012 dengue outbreak in Kolkata [15]. Another study by Jisamerin et al. demonstrates that fever was the primary symptom of DENV-positive patients, and most cases appeared from October to December in southern India [16]. One study modelled the role of anthropometric status on dengue in Colombian children in which DENV positivity was 60.8%. Waist circumference was positively correlated with seropositivity in females [17]. 

In India, all four serotypes (DEN-1, DEN-2, DEN-3, and DEN-4) of dengue were reported in the past. The present study found that the DENV-2 serotype was the dominant serotype among children with 91.66% positivity followed by a single case (8.33%) of DENV-3. As per the previous study on dengue serotypes, it is known that the re-infection from the same serotype in the future will self-infect-limiting without any warning sign but re-infection or co-infection from different serotypes of dengue in the same person may increase the severity of the disease due to the Antibody-dependent enhancement (ADE) phenomena [5]. Although DENV-3 was present in less number in this study, the chance of re-infection or co-infection with different serotypes in nearby future cannot be ruled out. Authors felt that the presence of two different serotypes in Uttarakhand may occur due to the highest footfall of pilgrims and tourists. This state is well famous for its education, religion, meditation, and yoga centres globally. Mishra et al. studied that ≤ 5-year children were most affected due to dengue fever and dengue 1-3 serotypes were present during 2009-12 in Uttar Pradesh [18]. A study from New Delhi during the monsoon of 2017 reported the prevalence of DEN-3 in their study population, followed by DEN-1 and 2, respectively [19]. Deval et al. from Uttar Pradesh reported the DEN-2 serotype in the population [20]. These states are near Uttarakhand and many locals travel in this state regularly which gives support to the theory of serotypes difference due to the travellers from different parts of the country. Studies from Indonesia and Pakistan reported that DEN-3 was dominant in children in 2014 and 2018, respectively [21,22]. However, in a study from Kenya during 2014-2017, all four serotypes were present in children, but the dominant serotype was DEN-2 [23]. While comparing the most common disease of childhood death in Cambodian children, dengue virus seroprevalence increased steadily with age, indicating constant dengue virus experience [24]. A study was conducted from 2002 to 2008, in which all four serotypes were found during the 2003 epidemic season. DEN-3 was predominant during 2004-2006 while DEN-2 was predominant in 2007. DEN-1 was found more in number during the 2008 epidemic [25]. Kalita et al. studied dengue in Rajasthan and reported the most common serotype was DEN-3 during 2014-2018 [6]. The frequent travelling from these areas to Uttarakhand may increase the chance of circulation of multiple serotypes of dengue in Uttarakhand.

Cross-reactivity between flaviviruses is very common in serological tests. This presents a significant challenge in the investigation of flavivirus diseases, particularly in areas where multiple viruses are endemic. The severity of cross-reactivity can vary depending on the species, serological tests used, and target antibody of detection. According to Endale et al., a systematic review of flavivirus cross-reactivity found up to 84% cross-reactivity in yellow fever virus with DENV and DENV with Zika virus, but only up to 7% in DENV with chikungunya virus and non-DENV flavivirus [26]. According to the Director General of Health Services of India, no cases of yellow fever have been registered in India due to its strict yellow fever vaccination programme [27], but Zika virus infection is spreading in this country. According to the Health Ministry of India, in 2021, 237 cases of the Zika virus were reported [28]. As a result, active surveillance of this virus, as well as dengue and chikungunya, is required. In this study only DENV was studied due to the limited resources and the low number of molecular studies due to the insufficient viral load was the main limitation of the study. However, the presence of the maximum number of the same serotypes in children gives a relief that the disease severity due to the ADE phenomena is limited but molecular studies on a larger scale for this arboviral infection during every dengue season will give the actual load of the disease with the circulating serotypes in this region.

## Conclusions

This is the preliminary study as the authors' best knowledge which reported the burden of dengue in children and screened all four serotypes of dengue and found the prevalent serotypes for consecutive three years in Uttarakhand. This study reported that the serotype-2 of dengue virus (DEN-2) was prevalent during the study which causes infection in children at tertiary care hospitals in northern India. These results will provide a foundation for future studies on the dengue virus in Uttarakhand and help further to understand the nature of the disease and genetic characteristics of the virus so that improved patient care management will imply. Further molecular studies on large sample sizes in this region, monitoring, and documentation of circulating serotypes from nearby states would be helpful to know the actual load of the disease and will provide knowledge of circulating/re-emerging serotypes of dengue in the future. 
